# Supplementary material for: Cysteines are critical determinants of spontaneous and seeded tau aggregation in cells
Source: Res Sq. 2026 Apr 23:rs.3.rs-9217102. Preprint. [Version 1] doi: 10.21203/rs.3.rs-9217102/v1 (PMC13131862; doi:10.21203/rs.3.rs-9217102/v1)
Supplement: 1 [file NIHPPrs9217102v1-supplement-1.pdf]

Supplementary Table 1. Cryo-EM data collection parameters for S320F$_{295\text{-}330}$ tau fragment fibrils.

**Data Collection**	
Microscope	Titan Krios
Acceleration Voltage (kV)	300
Detector	K3
Magnification	105,000x
Pixel size(Å/pix)	0.8332
Defocus range (μm)	-1.2 to -2.2
Total dose (e^-/Å)	62
Exposure time (s)	3.36
**Reconstruction**	
Micrographs	6,509
Box size(pix)	384
Total extracted segments	989,124
Number of segments after 2D	371,662
Number of segments after 3D	46,414
Symmetry imposed	C1
Helical rise (Å)	4.768
Helical twist (°)	-2.38
Crossover distance(Å)	330
Map-Sharpening B-factor(Å^2)	-101.704
Map resolution (Å; FSC =0.143)	3.7
**Atomic Model**	
Non-hydrogen atoms	1,722
RMSD bond (Å)	0.006
RMSD angles (°)	1.156
Molprobity score	2.16
Molprobity clash score	14.87
Rotamer outliers (%)	0.98
Cβ outliers (%)	0.00
Ramachandran plot(favored/allowed/outliers)	92.00, 8.00,0.00
CaBLAM outliers (%)	1.69

Supplementary Table 2: Parameters used during flow cytometry data collection for peptide and tauRD seeding assays in NK13 (tauRD P301S biosensors)

**Collected parameter**	Voltage	Excitation (nm)	Emission filter (nm)
Forward Scatter	100	488	
Side Scatter	380	488	
Acceptor, “mClover3”	220	488	525/50
Donor, “mCerulean3”	300	405	450/50
FRET, “AmCyan”	200	405	525/50

Supplementary Table 3: Parameters used during flow cytometry data collection of tauRD-mEOS3.2 expression system.

**Collected parameter**	Voltage	Excitation (nm)	Emission filter
Forward Scatter	100	488	-
Side Scatter	380	488	488/10 BP
Acceptor, “Alexa Fluor 488”	220	488	530/30
Donor, “mRuby3”	300	561	620/15
FRET, “PerCP”	300	488	695/40

Supplementary Table 4: Subjects studied.

Sample Name	ID	Age	Sex	Institution
AD_46121	46121	74	F	UTSW
AD_46879	46879	76	M	UTSW
AD_49341	49341	83	F	UTSW
AD_47833	47833	82	M	UTSW
AD_46090	46090	75	F	UTSW
AD_45408	45408	73	M	UTSW
AD_62579_Parietal	62579	71	M	WashU
AD_63007_Parietal	63007			WashU
AD_63870_Parietal	63870	81	F	WashU
AD_63870_Temporal	63870	81	F	WashU
CBD_48638	48638	64	M	UTSW
CBD_44193	44193	55	M	UTSW
CBD_46267	46267	67	F	UTSW
CBD_3950	3950	72	M	WashU
CBD_23302	22302	62	M	WashU
CTE_8240	9130	40	M	Boston University
CTE_6227	8408	49	M	Boston University
CTE_9284				Boston University
CTE_7865				Boston University
PSP_45460	45460	68	F	UTSW
PSP_47941	47941	69	M	UTSW
PSP_49017	49017	61	F	UTSW
PSP_48221	48221	67	M	UTSW

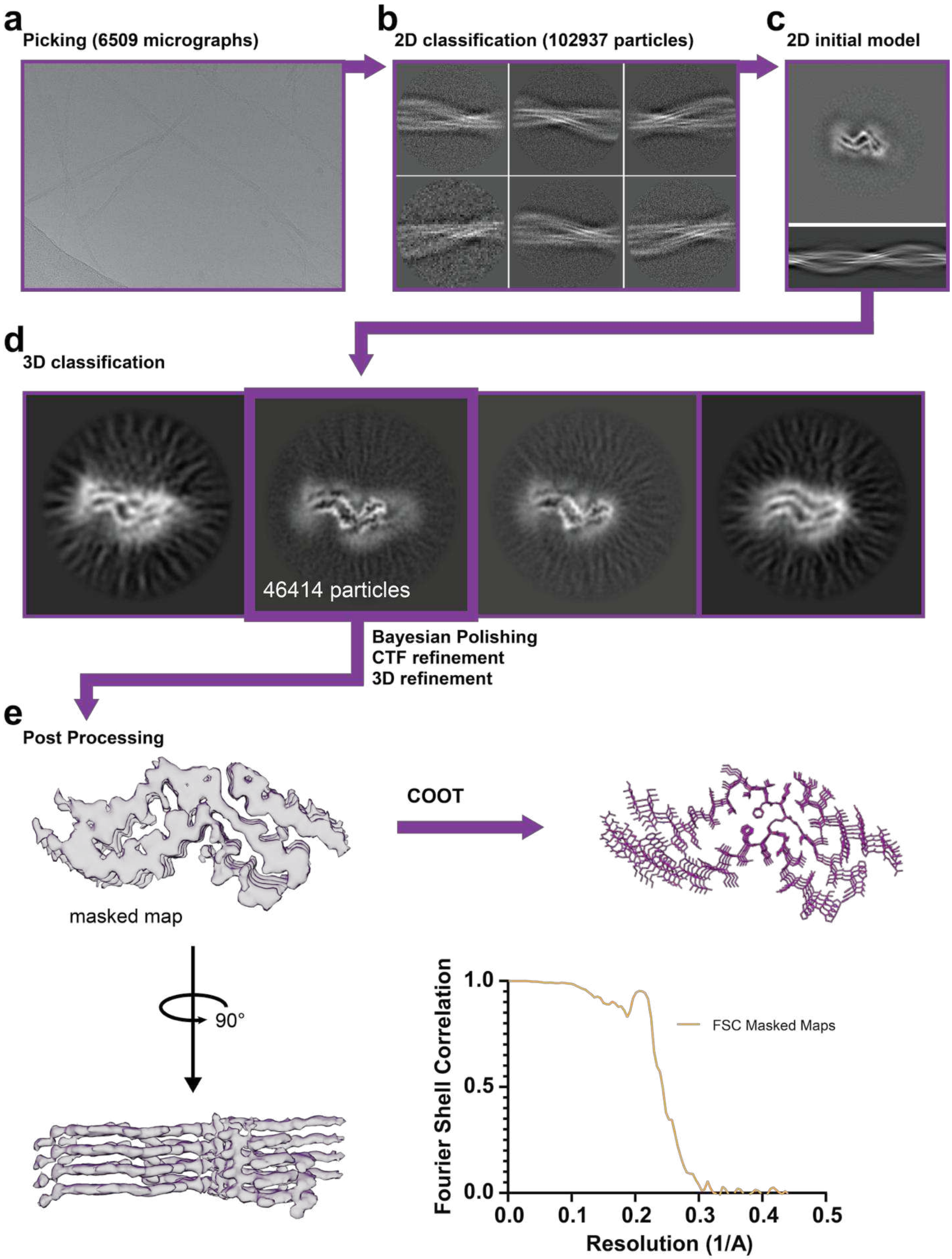

a
Picking (6509 micrographs)
b
2D classification (102937 particles)
c
2D initial model
d
3D classification
46414 particles
Bayesian Polishing
CTF refinement
3D refinement
e
Post Processing
masked map
COOT
90°
FSC Masked Maps
Fourier Shell Correlation
Resolution (1/A)
1.0
0.5
0.0
0.0
0.1
0.2
0.3
0.4
0.5

Supplementary Figure 1. Cryo-EM reconstruction of S320F$_{295\text{-}330}$ tau fibrils. Representative micrograph (**a**) and 2D class averages (**b**) were used to generate an initial 2D reference model (**c**). After particle selection (46,414 particles), multiple 3D classifications were performed (**d**), yielding a well-defined fibril reconstruction. The final 3D density map (**e**) was refined to high resolution, allowing atomic model building (top right) and visualization of fibril packing (bottom left inset). Fourier shell correlation (FSC) curves (bottom right) demonstrate the resolution and quality of the final reconstruction.

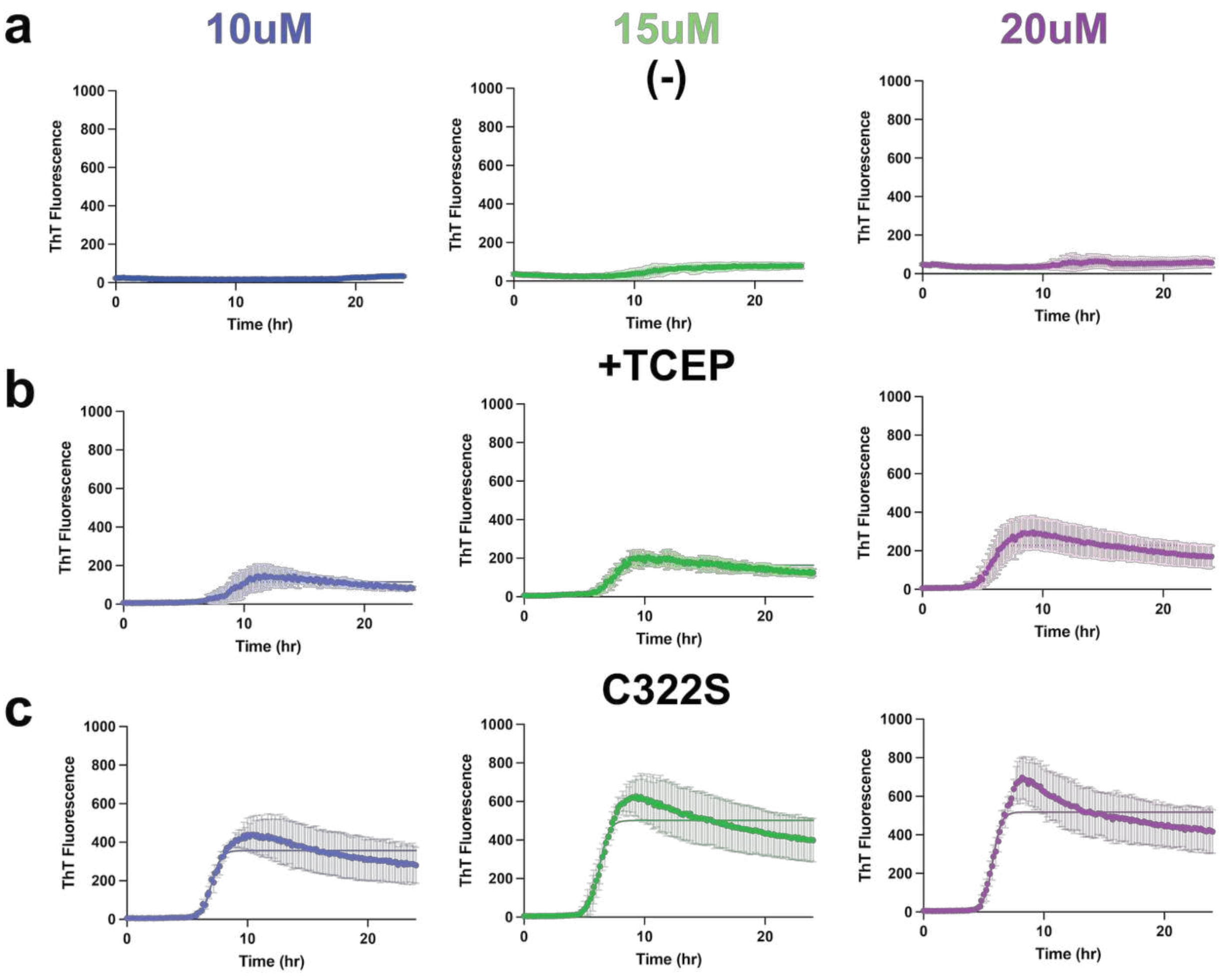

Supplementary Figure 2. **Kinetic traces of S320F$_{295\text{-}330}$ and mutant peptide aggregation.** ThT fluorescence aggregation kinetics on **(a)** S320F$_{295\text{-}330}$ (-), **(b)** bS320F$_{295\text{-}330}$ (TCEP), and **(c)** S320F$_{295\text{-}330}$ C322S at three different concentrations; 10 μM (blue), 15 μM (green) and 20 μM (magenta). All experiments were carried out as technical triplicates and each curve was fit to a non-linear regression model in GraphPad Prism to estimate $t_{1/2}$max and fluorescence amplitudes. The data is shown as an average with a standard deviation.

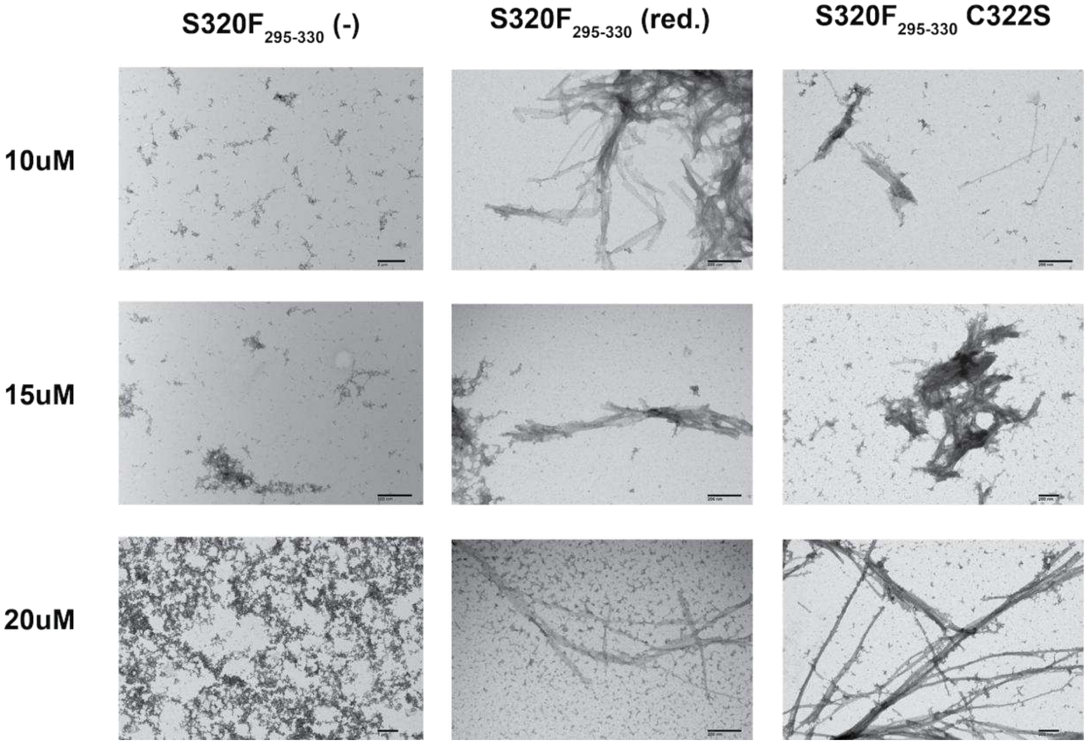

Supplementary Figure 3. **TEM verification of $S320F_{295–330}$ and C322 variant fibril samples.** TEM images at the endpoint of ThT fluorescence assay of $S320F_{295\text{-}330}$ without TCEP (-), with TCEP (red.) and $S320F_{295\text{-}330}$ C322S; 10 μM,15 μM and 20 μM (left to right). Scale bar represents 200 nm.

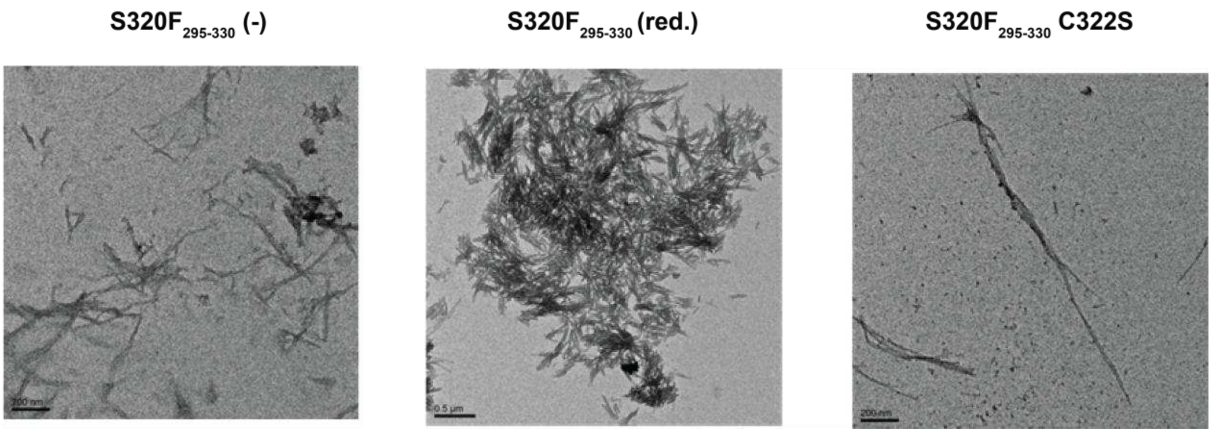

Supplementary Figure 4. Biochemical characterization and aggregation properties of S320F tau peptides and C322 variant. Negative-stain TEM images of fibrils formed by S320F$_{295–330}$ without TCEP (-), S320F$_{295–330}$ with TCEP (red.), and cysteine variant (C322S) of S320F$_{295–330}$. Scale bars 200-500 nm.

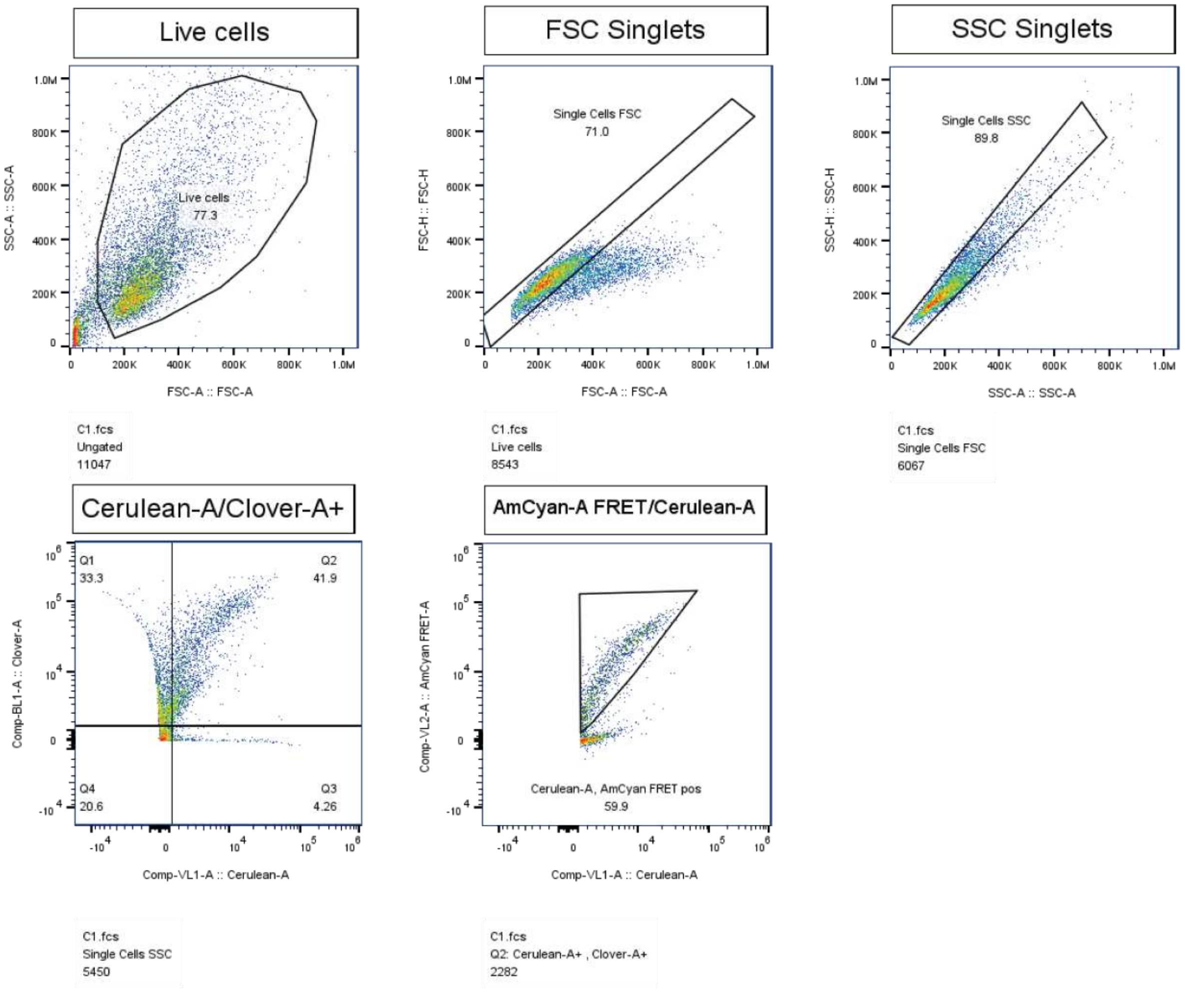

Supplementary Figure 5. **Gating strategy for seeding assay in tauRD P301S Cerulean/Clover biosensors with S320F peptides in various conditions and S320F tauRD, and S320F tauRD cysteine mutant fibrils** Gating strategy to extract live, single, mCerulean3 (cyan) and mClover3 (green) channel double-positive cells and expression level for FRET quantification between AmCyan-A FRET/mCerulean3-A.

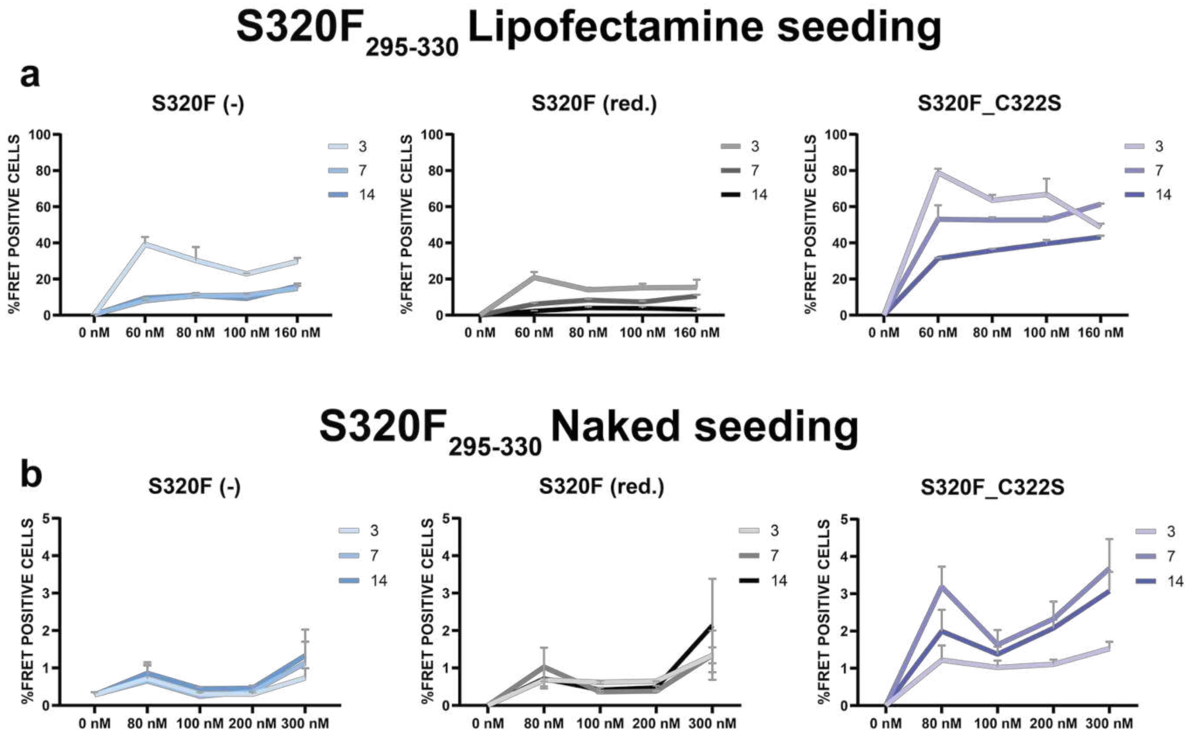

Supplementary Figure 6. Titration of S320F$_{295\text{-}330}$ and variant aggregates in cell seeding assays. (**a**) Lipofectamine-based seeding of fibrils S320F$_{295\text{-}330}$ under non-reducing conditions (-) (no TCEP), S320F$_{295\text{-}330}$ under reducing conditions (red.) (with TCEP) and S320F$_{295\text{-}330}$ C322S. Seeding was performed across samples pulled at 3, 7 and 14 days at 5 concentrations of fibrils (0, 80, 100, 200, and 300) nM for naked seeding, (0, 60, 80, 100, and 160) for lipofectamine seeding. (**b**) Naked-based seeding (direct) of fibrils (d) S320F$_{295\text{-}330}$ under non-reducing conditions (-) (no TCEP), S320F$_{295\text{-}330}$ under reducing conditions (red.) (with TCEP) and S320F$_{295\text{-}330}$ C322S. Seeding was performed across samples pulled at 3, 7 and 14 days at 5 concentrations of fibrils (0, 80, 100, 200, and 300 nM). Plots for the 3-day timepoint for S320F$_{295\text{-}330}$ (-), S320F$_{295\text{-}330}$ (red.), and S320F$_{295\text{-}330}$ C322S samples are colored light blue, grey and violet, respectively with the 7- and 14-day time points colored as darker shades of blue, grey and violet. Data is shown as averages of triplicates with SEM.

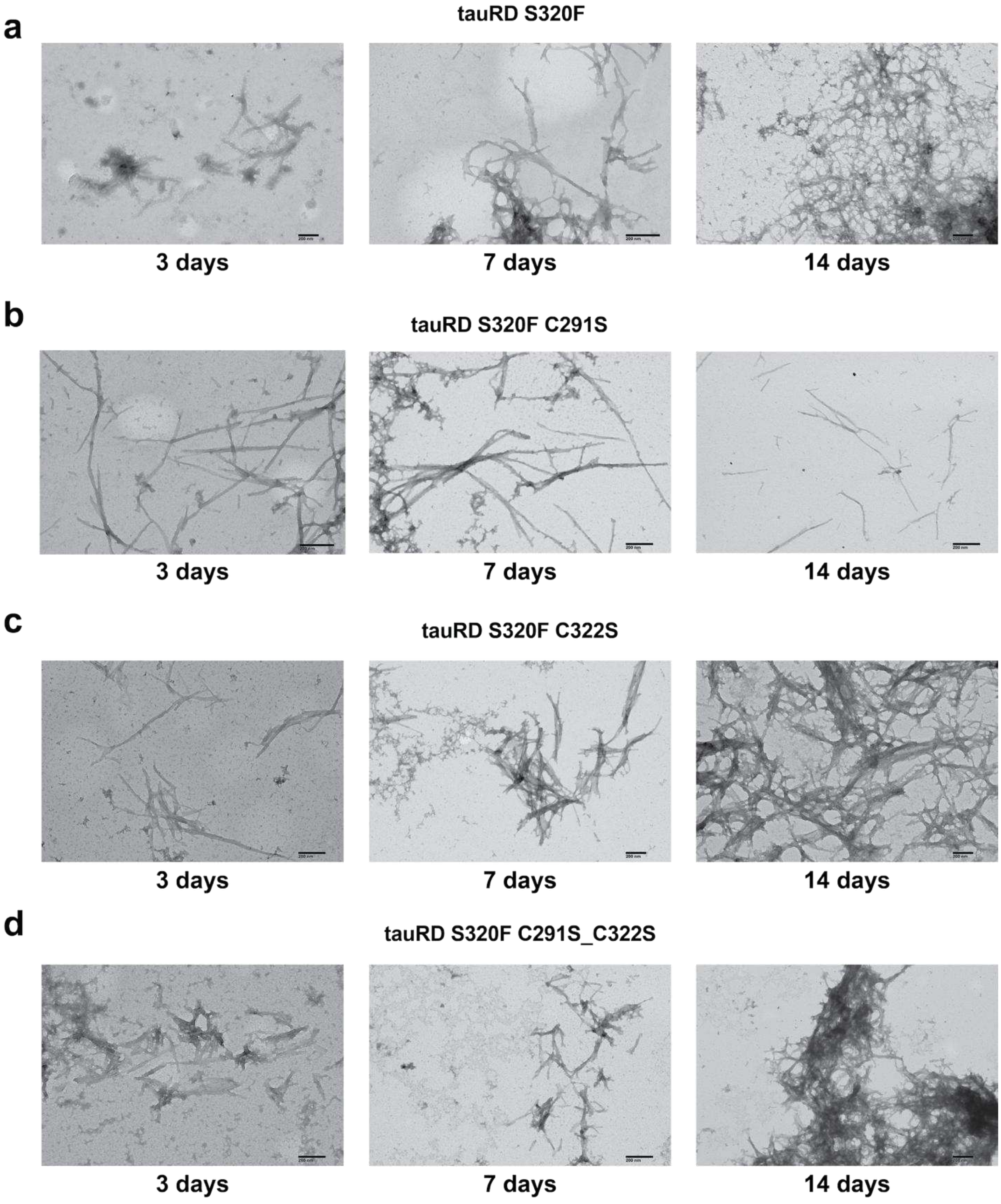

Supplementary Figure 7. **TEM-based confirmation of tauRD S320F, tauRD S320F C291S, tauRD S320F C322S and tauRD S320F C291S C322S aggregates.** TEM images of fibrils samples used for the lipofectamine and naked seeding experiments with three different time points; 3, 7 and 14 days (left to right). (**a**) tauRD S320F (TCEP), (b) tauRD S320F C291S (TCEP),

(**c**) tauRD S320F C322S (TCEP) and (**d**) tauRD S320F C291S_C322S (TCEP). Scale bar: 200 nm.

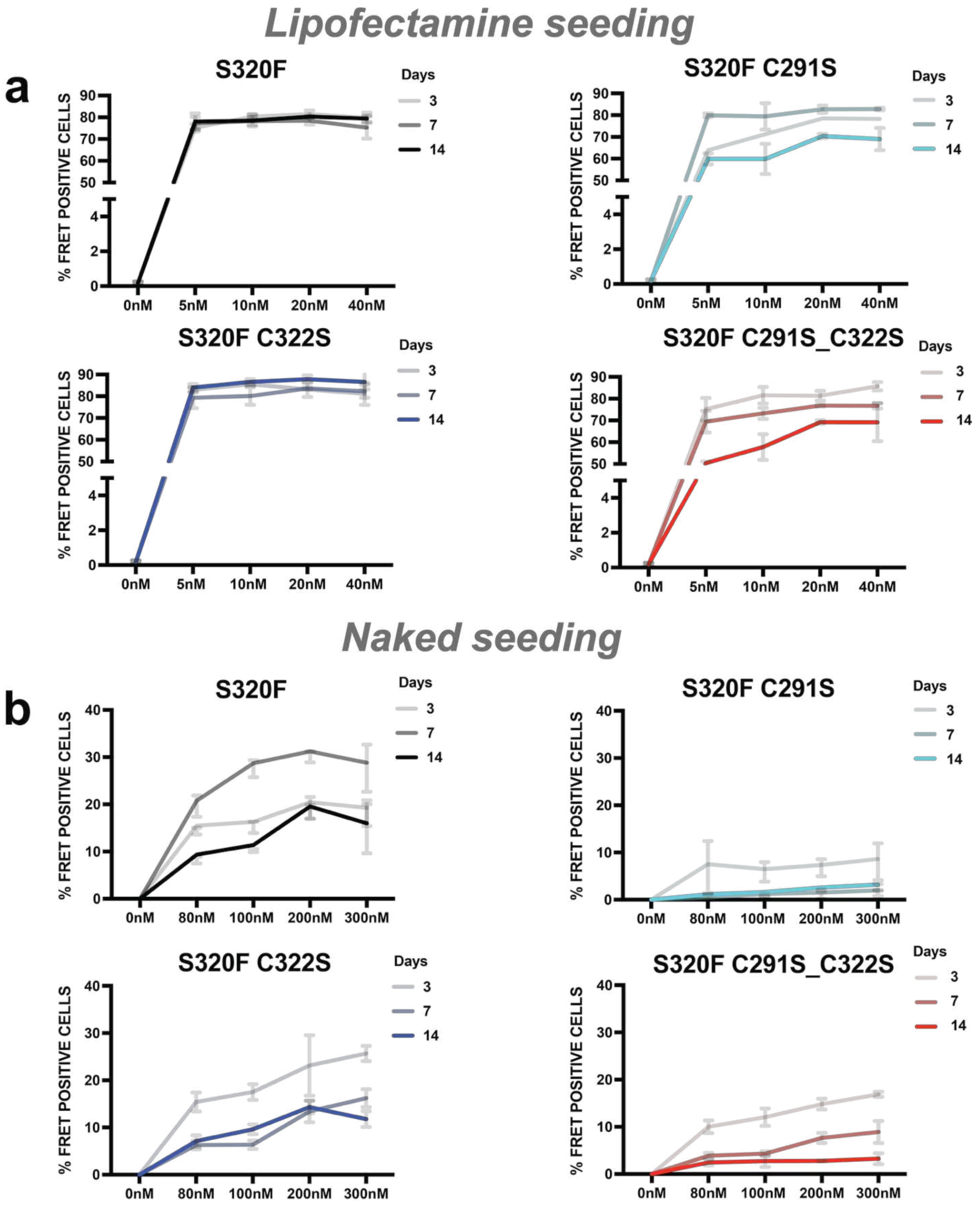

Supplementary Figure 8. Cell-based seeding activity of *in vitro* tauRD S320F and tauRD S320F cysteine mutant fibrils. Seeding was evaluated for samples incubated in vitro for 3, 7

and 14 days and delivered into tau biosensor cells with (lipofectamine) or without transfection reagent (naked seeding). tauRD S320F in vitro purified constructs consists of amino acid residues from 243 to 378 with a mutation at 320 positions with two cysteines at positions C291 and C322. Seeding experiments were carried out with fibrils at three time points (3, 7 and 14 days) using naked and lipofectamine mediated seeding. (**a**) Percent FRET positivity from lipofectamine-mediated seeding activity of fibrils formed from tauRD S320F (TCEP) (black), S320F C291S (cyan), S320F C322S (blue) and S320F C291S_C322S (red). Preformed fibrils used are 0, 5, 10, 20, and 40 nM concentrations incubated with lipofectamine. 0nM is lipofectamine (vehicle only control). (**b**) Percent cell FRET positivity from naked seeding activity (in the absence of transfection reagents) of fibrils formed by incubating tauRD S320F (black), S320F C291S (cyan), S320F C322S (blue) and S320F C291S_C322S (red) preformed fibrils at 0, 80, 100, 200 and 300 nM concentrations with cells. All data is in triplicates tittered from 80nM to 300nM. All the values plotted are averages with SEM. Data is shown as averages of triplicates with SEM.

.

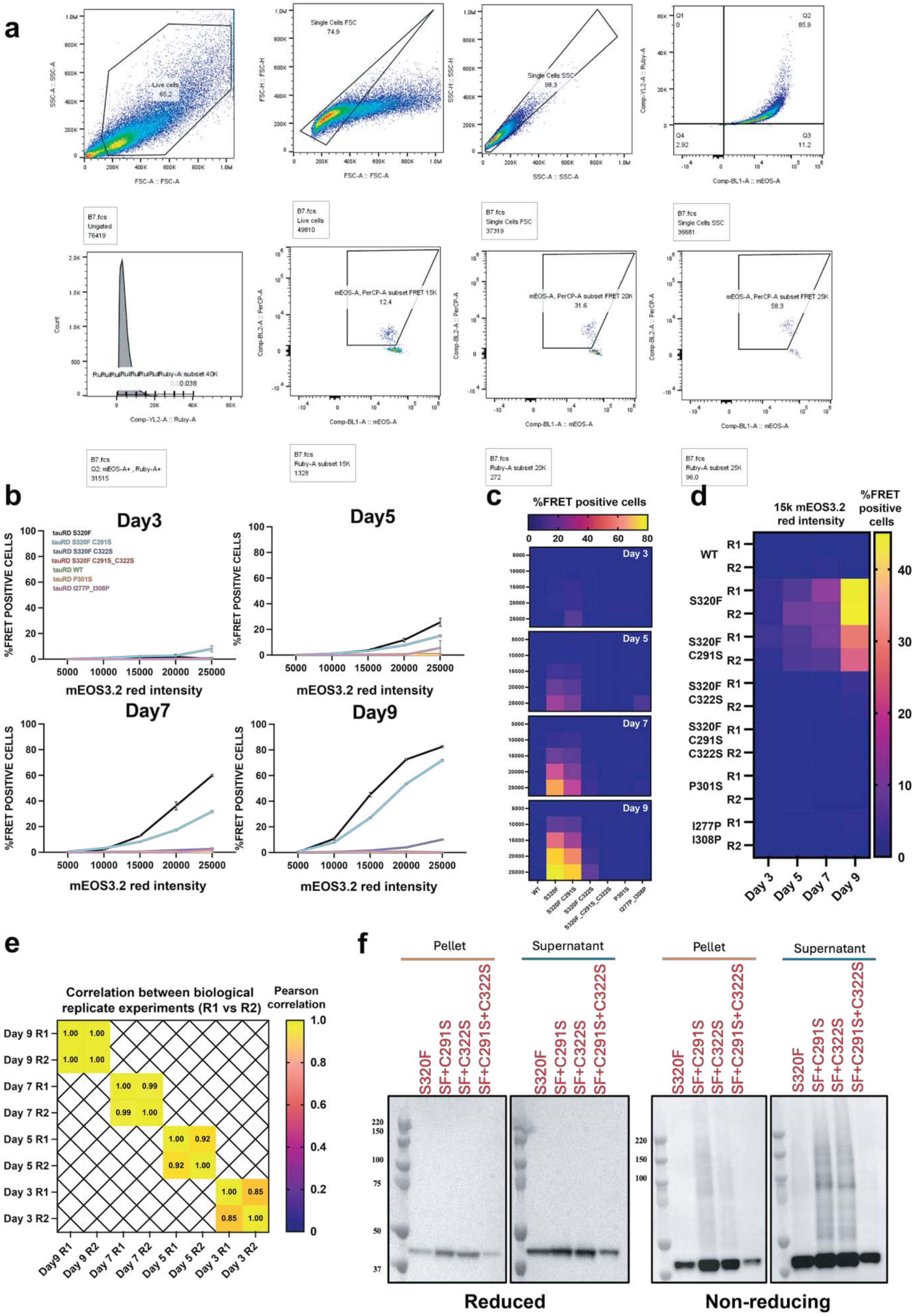
a
b
Day3
Day5
Day7
Day9
%FRET POSITIVE CELLS
mEOS3.2 red intensity
tauRD S320F
tauRD S320F C291S
tauRD S320F C322S
tauRD S320F C291S_C322S
tauRD WT
tauRD P301S
tauRD I277P_I308P
c
%FRET positive cells
d
15k mEOS3.2 red intensity
%FRET positive cells
e
Correlation between biological replicate experiments (R1 vs R2)
Pearson correlation
f
Pellet
Supernatant
S320F
SF+C291S
SF+C322S
SF+C291S+C322S
Reduced
Non-reducing

Supplementary Figure 9. Time course to characterize S320F tauRD and cysteine mutant spontaneous aggregation in cells. (a) Representative flow cytometry gating strategy and distribution plots used to quantify aggregate-positive cells, showing sequential gating, fluorescence intensity thresholds, and population shifts upon spontaneous aggregation. **(b)** Time-course quantification of aggregate accumulation, plotted as mean signal intensity on x axis and percentage of FRET positive cells on y-axis across multiple days (Days 3, 5, 7, and 9). Data is shown as averages of three technical replicates with standard error of mean . **(c)** Heat maps summarizing spontaneous in cell aggregation kinetics across tauRD S320F and tauRD S320F cysteine mutants and time points, highlighting progressive increases in percentage of FRET positive cells as a function of red fluorescence signal intensity (5k, 10k, 15k, 20k, 25k) in arbitrary units. Data is shown as average from three technical replicates. Heatmaps are colored in plasma, with yellow High FRET and blue Low FRET. (**d**) Comparison of replicate 1 (R1) and replicate 2 (R2), biological replicates for spontaneous aggregation of tauRD S320F, tauRD S320F C291S, tauRD S320F C322S, tauRD S320F C291S_C322S, tauRD P301S, tauRD WT and tauRD I277P_I308P measured at days 3, 5, 7 and 9. Data is shown as a heat map comparing percentage of cells with FRET positive aggregates (from 15k $mEOS3.2_{red}$ intensity) for each construct in R1 and R2. Data is shown as an average across three technical replicates. Heat map is colored in plasma with high and low percentage of cells with FRET positive cells colored yellow and blue. (**e**) Pearson correlation of percentage of FRET positive cells (at 15k $mEOS3.2_{red}$ intensity) for replicate 1 (R1) and replicate (R2) across all constructs at days 3, 5, 7 and 9. Data is shown as a heat map and colored from yellow for high correlation and red for low correlation. **(f)** Biochemical validation of assemblies immunoblotting against tau of pellets and supernatants of lysates from day 9 after transduction resolved on SDS-PAGE under reducing and non-reducing conditions.

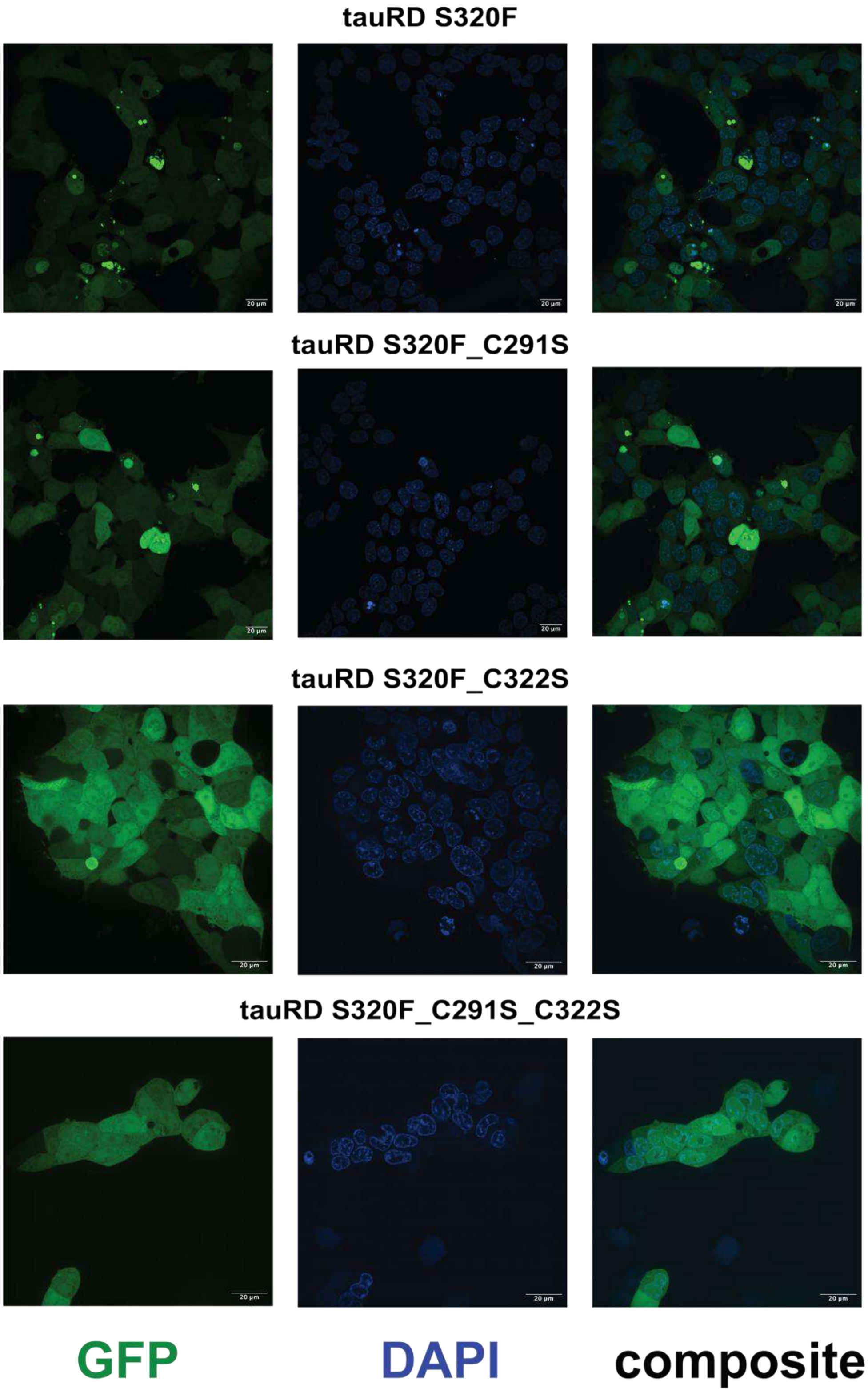

Supplementary Figure 10. **Effects of cysteine mutations on spontaneous aggregation of tauRD S320F in cells.** Representative fluorescence microscopy images of cells expressing repeat domain (tauRD) S320F and cysteine variants. Rows show tauRD S320F (top), tauRD

S320F C291S, tauRD S320F C322S, and tauRD S320F C291S_C322S (bottom). Columns display GFP fluorescence (left; tau aggregation reporter), DAPI nuclear staining (middle), and merged composites (right). Robust GFP-positive inclusions are observed for tauRD S320F and the C291S variant, indicating efficient aggregation and seeding in cells. By contrast, mutation of C322S blocks and double cysteine mutant (C291S_C322S) strongly suppress inclusion formation. Scale bars, 20 μm. Cells were imaged at 9 days post transduction, except for tauRD S320F and tauRD S320F C291S which were imaged at day 3. All images were processed using Fiji.

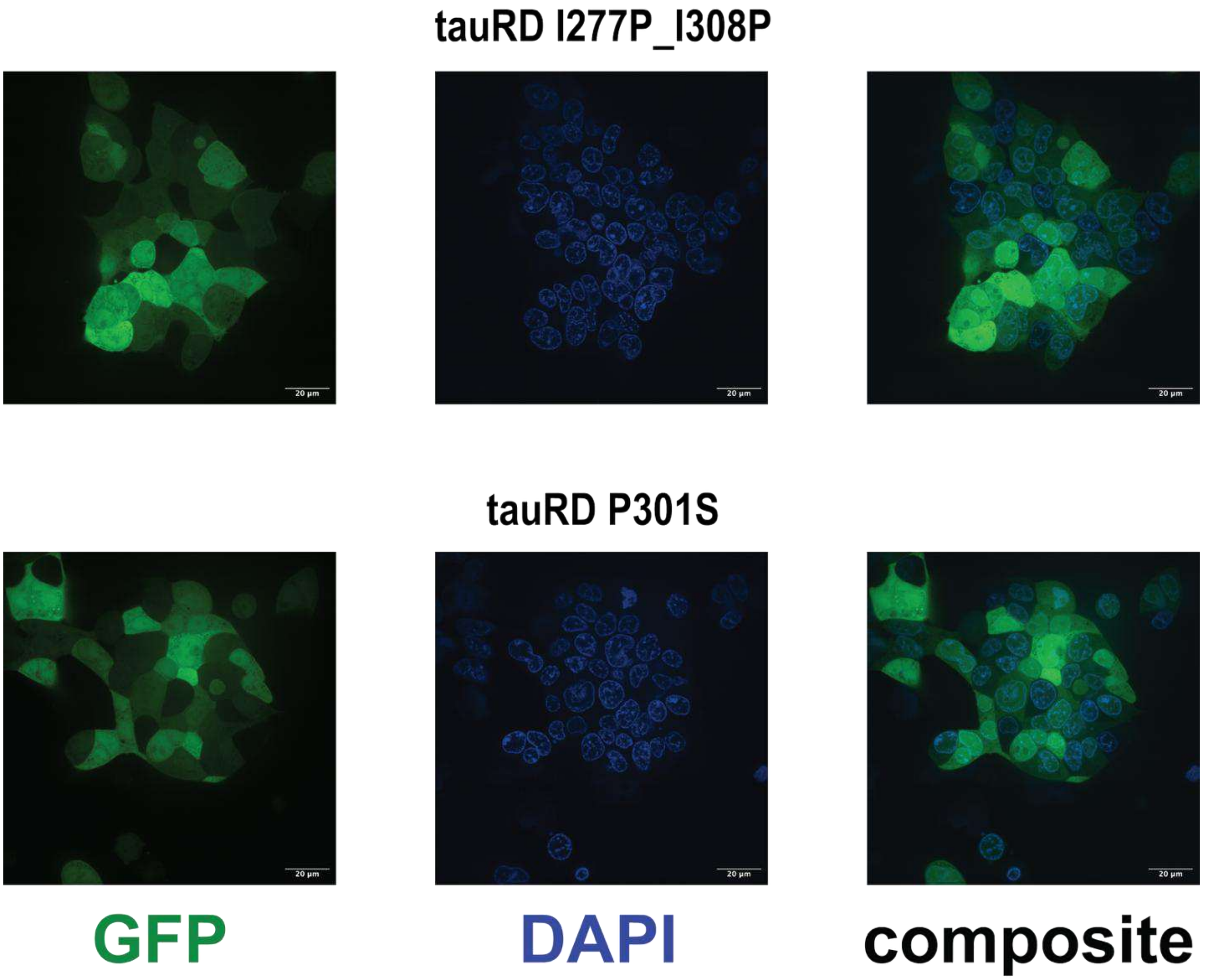

Supplementary Figure 11. Cellular aggregation phenotypes of control I277P_I308P and P301S tauRD constructs. Representative fluorescence microscopy images of cells expressing tau repeat domain (tauRD) variants encoding either the double proline mutation I277P_I308P (top row) or the frontotemporal dementia-associated mutation P301S (bottom row). GFP fluorescence (left) reports tauRD, DAPI (middle) labels nuclei, and merged composite images (right) show the spatial relationship between diffusely localized tauRD and cell nuclei. Scale bars, 20 μm. Cells were imaged at 9 days post transduction. All images were processed using Fiji.

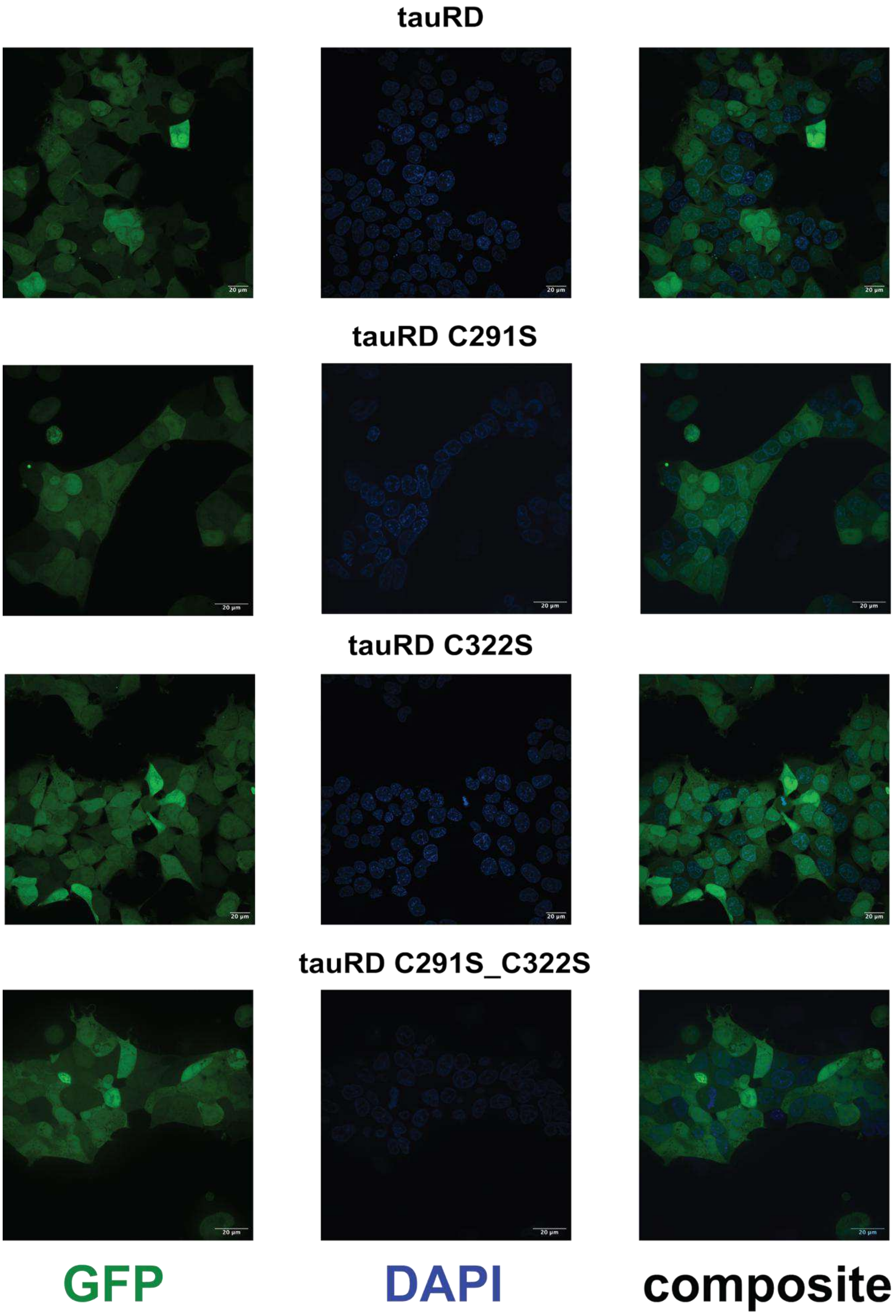
tauRD
20 µm
tauRD C291S
tauRD C322S
tauRD C291S_C322S
GFP
DAPI
composite

Supplementary Figure 12. Cysteine-dependent modulation of tauRD inclusion formation. Representative fluorescence microscopy images of cells expressing the tau repeat domain (tauRD) and cysteine variants. Rows show tauRD (WT) (top), tauRD C291S, tauRD C322S, and the double mutant tauRD C291S_C322S (bottom). GFP fluorescence (left) reports tauRD inclusion formation, DAPI staining (middle) labels nuclei, and merged composite images (right) show the cellular distribution of tauRD relative to nuclei. WT tauRD, tauRD C291S, tauRD C322S and tauRD C291S_C322S do not form inclusions over the course of this experiment. These images demonstrate that native cysteine residues modulate tauRD self-association and aggregation behavior in a cellular context. Scale bars, 20 μm. Cells were imaged at 9 days post transduction. All images were processed using Fiji.

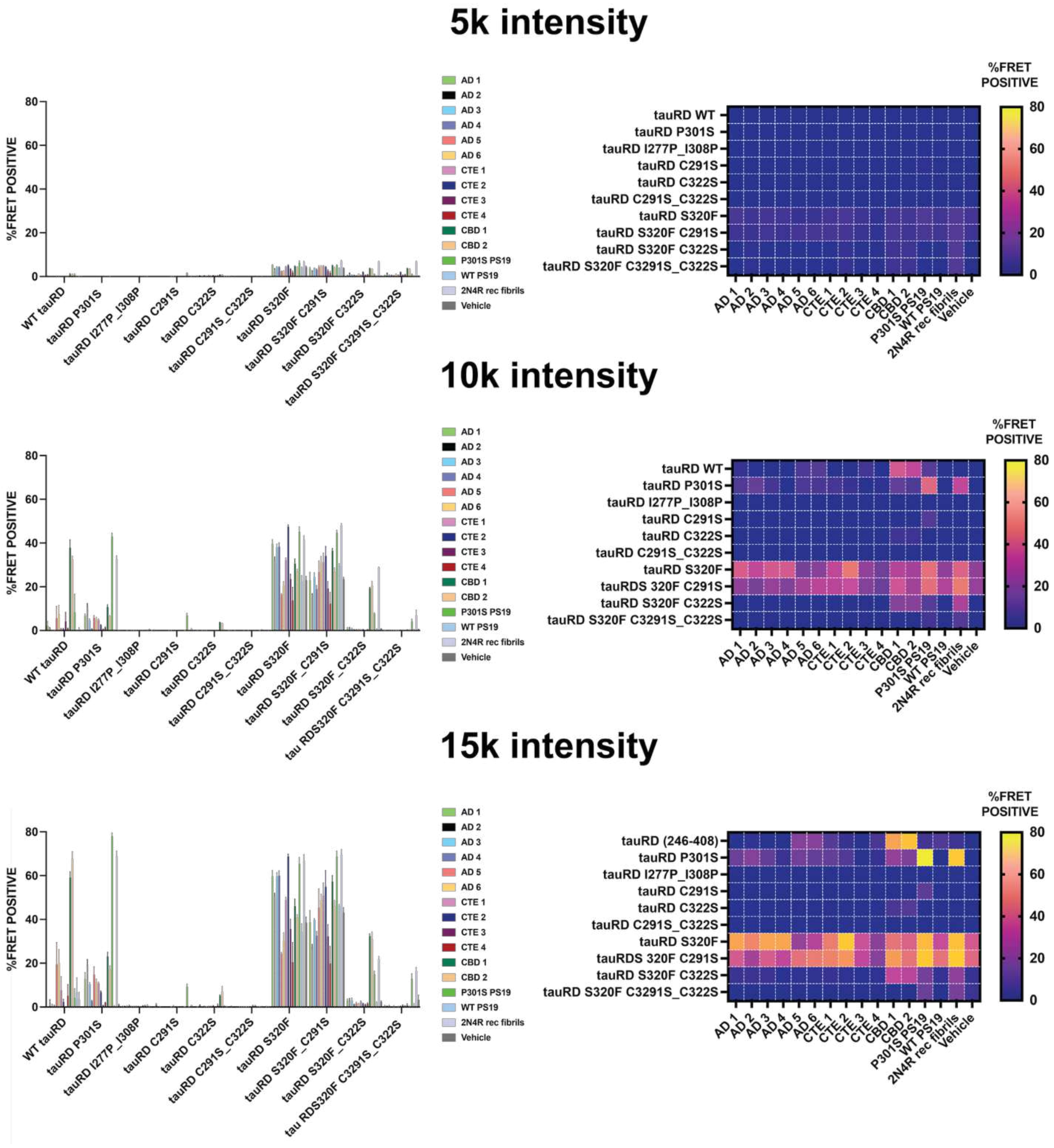

Supplementary Figure 13. Seeded aggregation of cysteine mutants in WT tauRD and tauRD S320F. Analysis comparing WT tauRD and S320F tauRD constructs encoding cysteine mutations, illustrating differential seeded aggregation propensities. Spontaneous aggregation shown separately for WT tauRD or tauRD S320F with cysteine mutants at 5K (top), 10K (middle), 15K (bottom) at mEOS3.2$_{red}$ intensity. Cells were seeded with tauopathy tissues including AD, CTE, CBD, PS19 and PS19 control animal tissues and recombinant 2N4R tau heparin

aggregates. Experiments were performed in triplicate with data shown as average with standard error of mean in bar plot representation (left) and averages as heatmaps shown in plasma color scheme with low and high % of FRET positive cells colored purple and yellow, respectively (right).

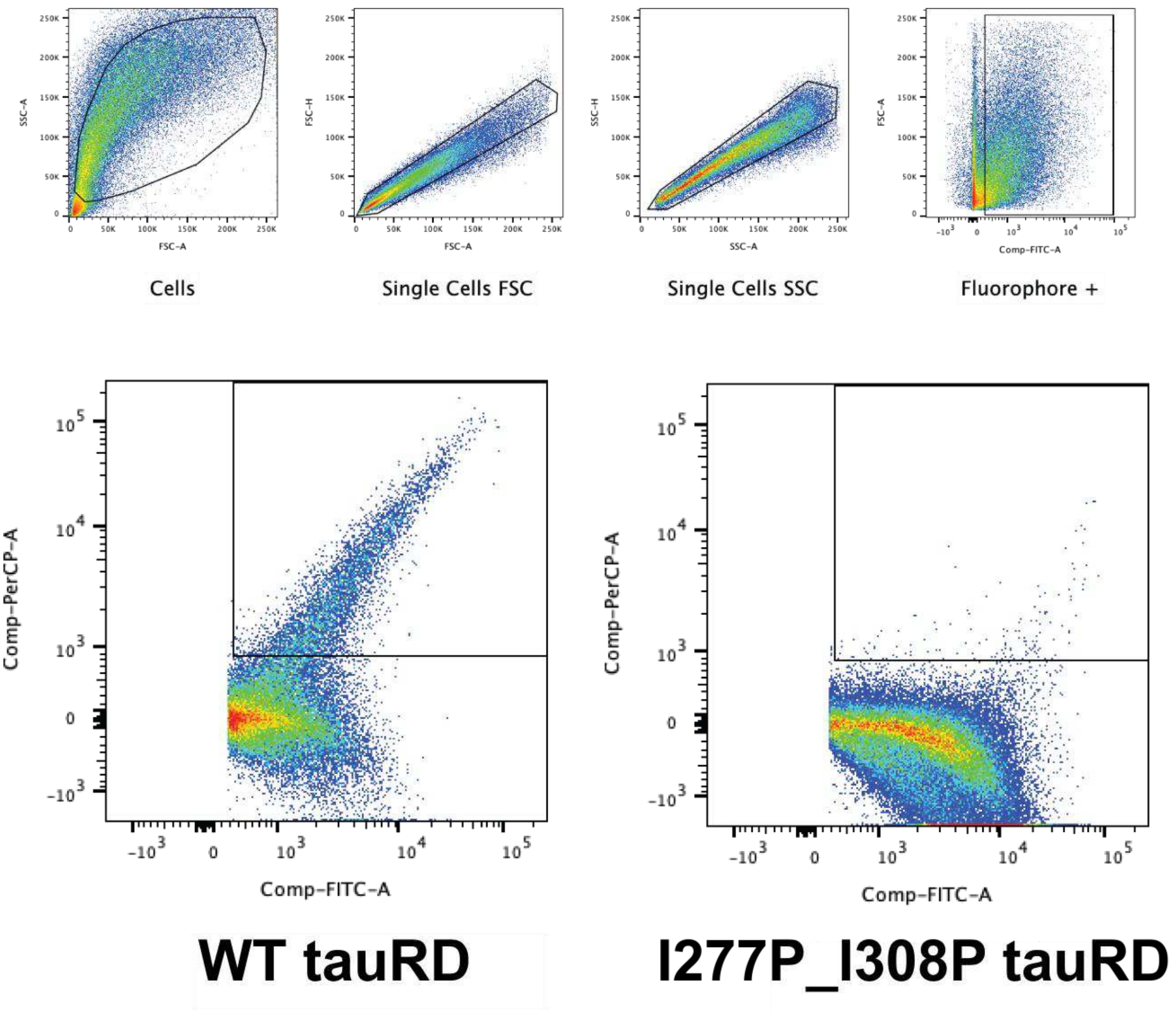

Supplementary Figure 14. Gating Strategy for Alanine Scanning mutagenesis-based seeding assay with AD, CBD and PSP tauopathy tissues. (a) Representative flow cytometry gating strategy and distribution plots used to quantify aggregate-positive cells, showing sequential gating, fluorescence intensity thresholds, and population shifts upon seeded aggregation. Example Gating strategies highlighting populations of FRET positive cells for WT tauRD (bottom left) and negative control I277P_I308P tauRD mutant (bottom right).

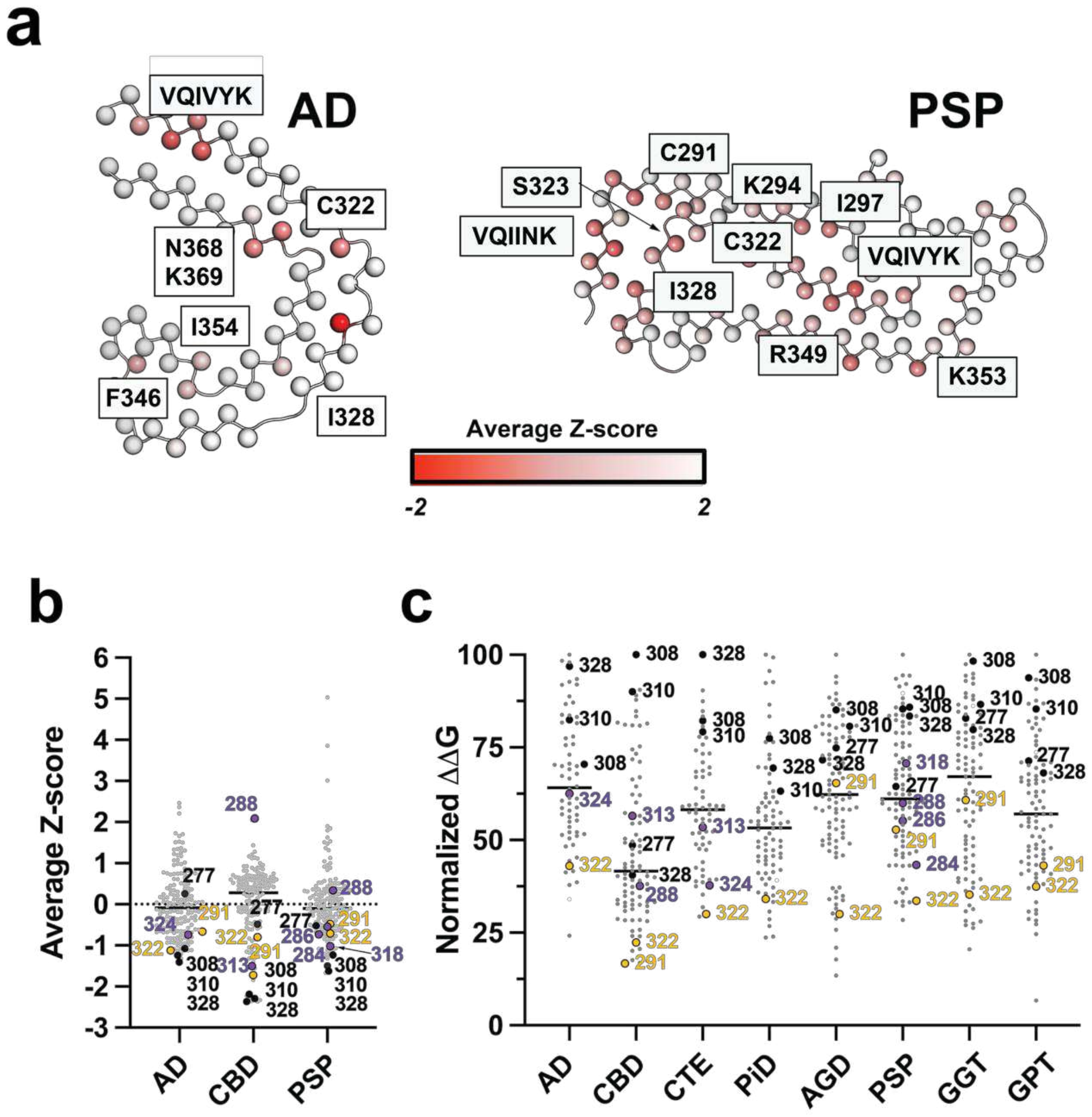

Supplementary Figure 15. Disease- and seed-specific contributions of tau cysteine residues to aggregation energetics and seeding activity. (a) Residue-level maps of average Z-scores mapped onto tau fibril structural models highlighting positions that contribute most strongly to aggregation propensity in Alzheimer's disease (AD, left) and progressive supranuclear palsy (PSP, right). Core amyloid motifs (VQIVYK and VQIINK) and key residues, including C291 and C322, are annotated. Color scale indicates relative stabilization or destabilization (average Z-score). **(b)** Distribution of average Z-scores for tau repeat-domain residues across disease contexts, emphasizing the prominent contributions of C291 and C322 relative to surrounding positions. **(c)** Normalized aggregation/seeding activity for alanine substitutions across multiple tauopathy-derived seeds, demonstrating disease-dependent sensitivity to mutations at C291 and C322 compared with other residues. Together, these analyses identify conserved cysteines as

major determinants of tau aggregation energetics and seeded assembly, with effects comparable to canonical amyloid-forming motifs.

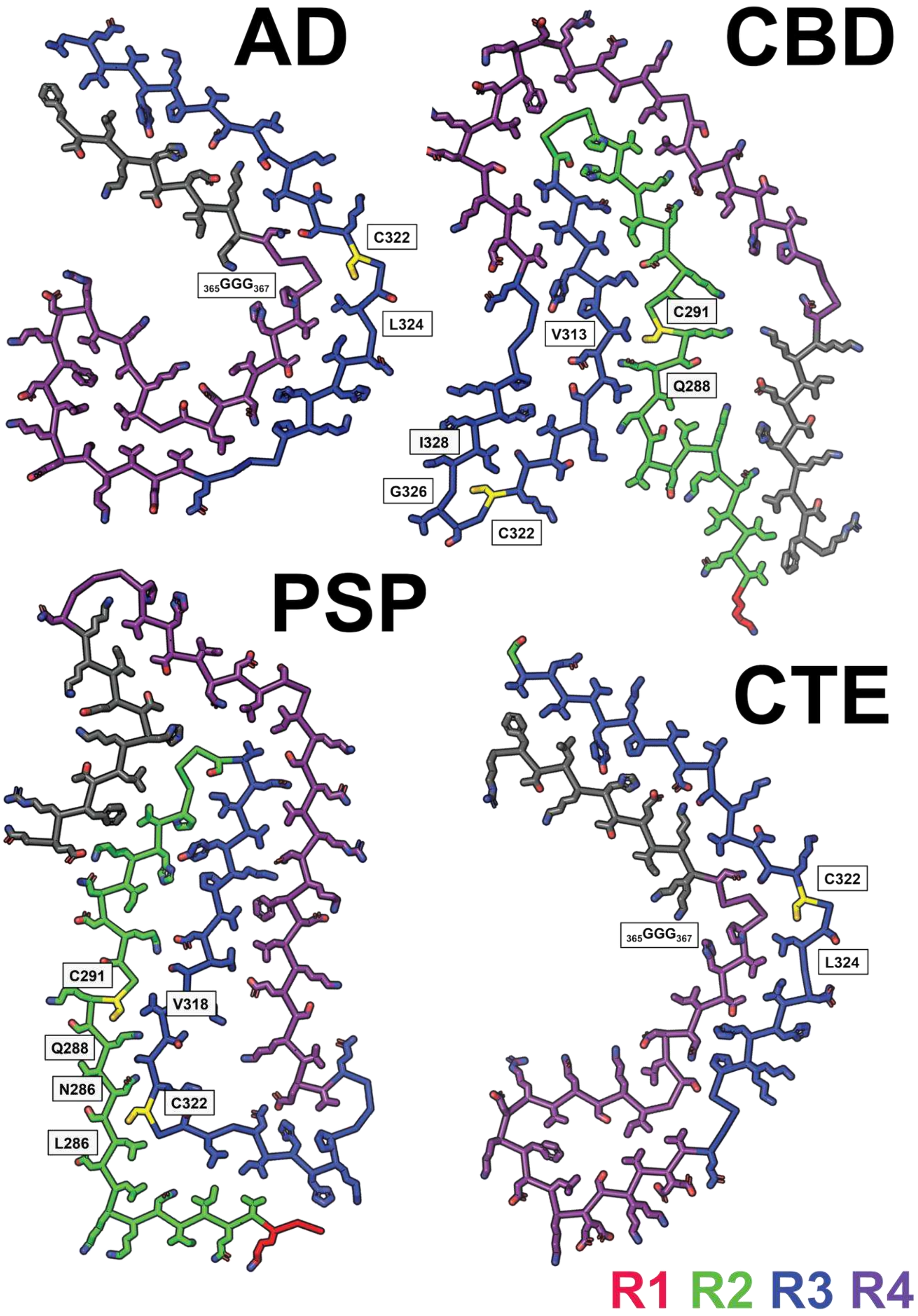

AD
C322
365GGG367
L324
CBD
C291
V313
Q288
I328
G326
C322
PSP
C291
V318
Q288
N286
C322
L286
CTE
C322
365GGG367
L324
R1 R2 R3 R4

Supplementary Figure 16. Disease-specific tau fibril architectures highlight distinct positioning of cysteine residues and amyloidogenic motifs. Atomic models of tau repeat-domain fibrils derived from brains of individuals with Alzheimer's disease (AD) (top left), corticobasal degeneration (CBD) (top right), progressive supranuclear palsy (PSP) (bottom left), and chronic traumatic encephalopathy (CTE) (bottom left). Individual tau repeats are color coded (R1, red; R2, green; R3, blue; R4, purple), with key residues and motifs annotated. Residues are shown as sticks and colored by repeat domain. Cysteine residues C291 and C322 are highlighted in yellow, illustrating their burial or local interactions in each fibril structure.

Supplementary Files

This is a list of supplementary files associated with this preprint. Click to download.

- SourceData.xlsx
- D1000297920valreportfullP1.pdf
